# Effectiveness of Factor XIII Infusion in Treatment of Refractory Ureteral Leakage after Kidney Transplantation

**DOI:** 10.1155/2020/1780760

**Published:** 2020-07-16

**Authors:** Ryoichi Maenosono, Tomohisa Matsunaga, Hajime Hirano, Hayahito Nomi, Shunri Taniguchi, Yuya Fujiwara, Koichiro Minami, Hirofumi Uehara, Teruo Inamoto, Haruhito Azuma

**Affiliations:** ^1^Department of Urology, Osaka Medical College, Osaka, Japan; ^2^Department of Urology, Osaka Saiseikai Nakatsu Hospital, Osaka, Japan; ^3^Department of Urology, Nozaki Tokushukai Hospital, Osaka, Japan

## Abstract

Despite the evolution of transplantation techniques, urological complications are common and result in loss of graft. We report the case of a 57-year-old man who developed continuous urine leakage despite pyeloureteral neoanastomosis and stenting after kidney transplantation from his dizygotic twin. Suspecting ureteral leakage, we performed pyeloureteral neoanastomosis using his native right ureter and a ureteral stent 5 days after the kidney transplant. However, urine leakage continued for several days. Because the plasma factor XIII level decreased to 48%, we administered factor XIII products (Fibrogammin P; CSL Behring, King of Prussia, PA) after the surgery. Although its utility and safety in patients with renal failure and/or transplantation are unclear, urine leakage stopped after the infusion of fibrogammin without any side effects. This is the first case report of the use of factor XIII for refractory urine leakage after kidney transplantation. Although further studies are needed, administration of factor XIII products could be one option for refractory urine leakage after transplantation.

## 1. Introduction

Urological complications after kidney transplantation are common and could lead to severe urinary tract infections or even early loss of renal graft [1]. Generally, periureteral tissues contain feeding vessels, and overdissecting the tissues around the ureter could cause necrosis, leading to ureteral leakage [2]. However, a ureteral stent is usually placed to secure the anastomosis and prevent ureteral obstructions [3]. Although well known, the extent to which these clinical approaches are needed remains unclear. The urological complications that develop after kidney transplantation are usually treated by stenting and/or surgical reconstruction, depending on the severity or the protocol in each institute.

In this report, we describe the case of a patient who experienced refractory ureteral leakage despite pyeloureteral anastomosis and ureteral stent placement. The patient demonstrated a decreased plasma coagulation factor XIII (FXIII) level, and the leakage improved after infusion of human plasma coagulation FXIII products (Fibrogammin P; CSL Behring, King of Prussia, PA). This is the first report on the utility and safety of FXIII products in transplant recipients.

## 2. Case Presentation

**Figure 1:**
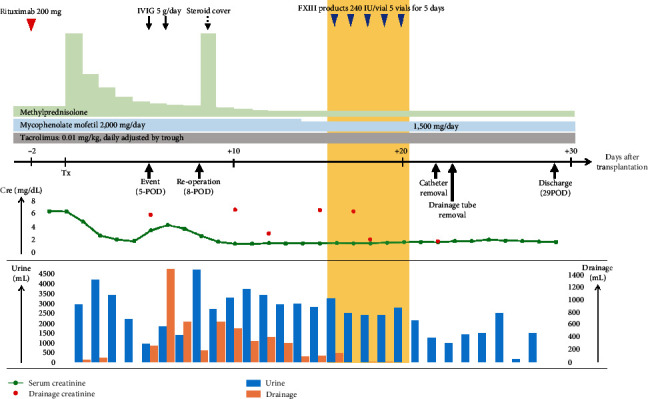
Perioperative clinical course. Cre: creatinine; FXIII: factor XIII; IVIG: intravenous immunoglobulin; POD: postoperative day.

A 57-year-old male patient was admitted to our department for kidney transplantation. He was suspected to have diabetes when he was 50 years old, because of an elevated hemoglobin A1c (HbA1c) level at 8.7%. Four years later, when he was referred to our hospital for kidney transplantation, his serum creatinine level was elevated, while his HbA1c level had decreased to 4.8%. When his serum creatinine level was elevated to approximately 6 mg/dL, he consented to undergo preemptive kidney transplantation. The donor was his 57-year-old female dizygotic twin; however, non-donor-specific antibodies were detected. Immunosuppression was performed using rituximab (200 mg/body), tacrolimus (0.1 mg/kg/day; adjusted by trough levels of 6-8 ng/mL), mycophenolate mofetil (2,000 mg), and methylprednisolone (20 mg/day before transplantation; 500, 250, and 125 mg/day on the day of transplantation, day 1 after the operation, and day 2 after the operation, respectively). The transplant procedure was completed without any complications, with a cold ischemia time of 2 hours and 23 minutes. Diuresis began 5 minutes after declamping. The Lich-Gregoir method was adopted without the use of a ureteral stent [3] (Figure 1).

**Figure 2:**
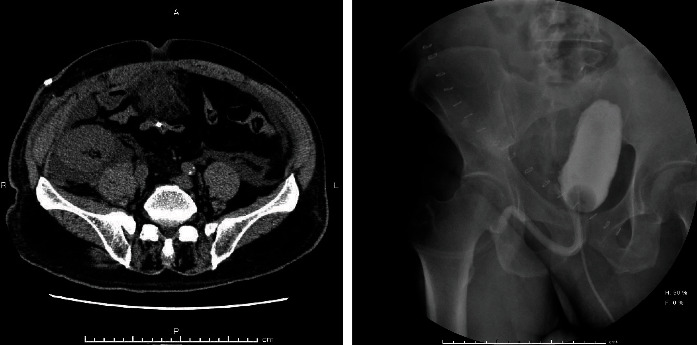
On postoperative day 5, fluid collection around the graft kidney is detected on computed tomography. Further, this fluid is not derived from the rupture of the anastomosis. Cystography reveals no leakage from the anastomosis.

**Figure 3:**
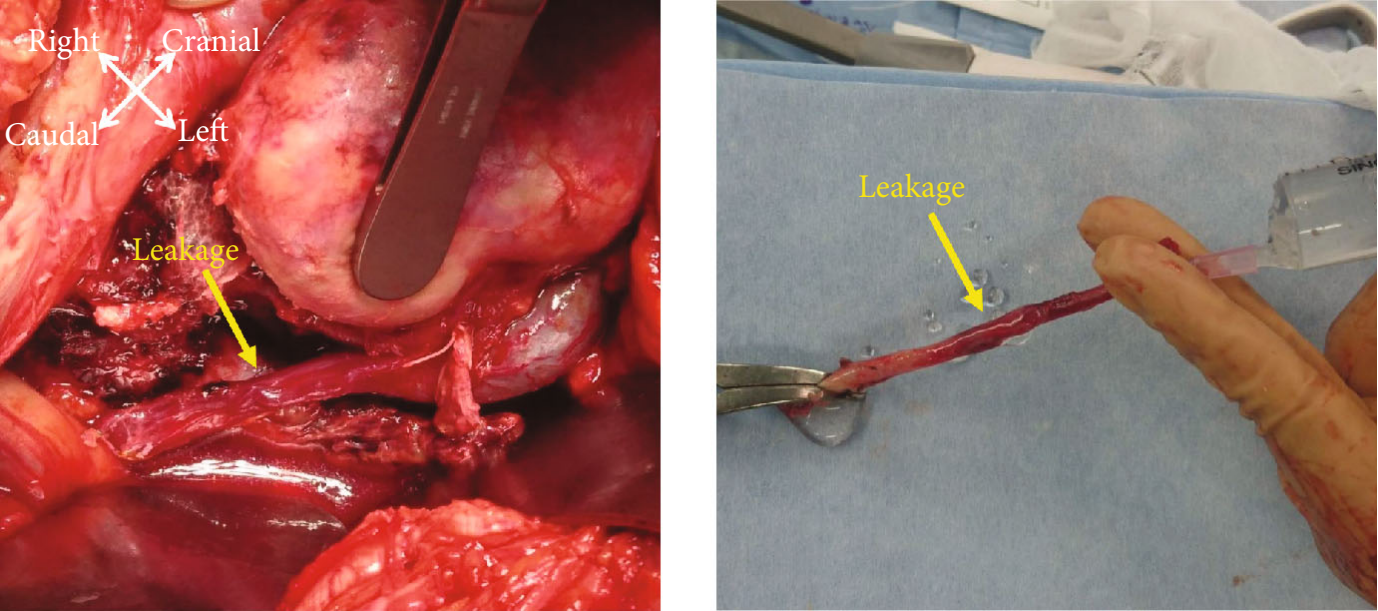
On surgical examination, urine is seen overflowing from the incision, and increased urine is seen around the graft kidney leaking from a tiny hole in the middle portion of the ureter.

**Figure 4:**
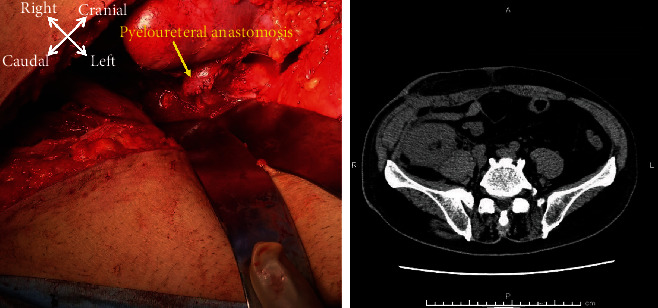
After native nephrectomy, reconstruction is performed with pyeloureteral anastomosis. A running suture is placed in a watertight manner with 3-0 vicryl after placing a double-J ureteral stent. After surgery, computed tomography revealed no residual urine around the graft.

After the surgery, the urine output and serum creatinine levels improved until postoperative day 4 (POD 4). On POD 5, he complained of sudden abdominal pain with decreased urine output. Computed tomography (CT) revealed fluid collections in the right postperitoneal cavity (Figure 2). In anticipation of a potential ureteral complication, retrograde stenting was attempted but was not accomplished owing to edema of the ureteral orifice. No leakage of contrast medium was seen on cystography, indicating a suspected ureteral rupture. On surgical examination performed on POD 8, we noted a small perforation in the middle portion of the right ureter (Figure 3). Further, pyeloureteral neoanastomosis was performed using 3-0 vicryl with a ureteral stent and drainage tube (Figure 4).

**Figure 5:**
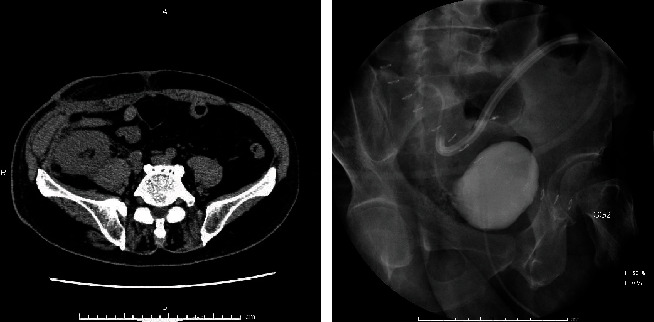
On postoperative day 14, urine leakage is not obvious on cystography. Further, computed tomography revealed no signs of leakage after removal of the drainage tube.

The pyeloureteral neoanastomosis showed persistent urine leakage along with a higher creatinine level than that in the serum for seven days after the repair; further, the coagulation FXIII level decreased to 48%. Based on the suspicion of a fistula, fibrogammin (240 IU/vial, 5 vials/day; CSL Behring, King of Prussia, PA) was administered for 5 days, which led to reduced drainage. Because there were no signs of leakage (Figure 5), the ureteral catheter and drainage tube were removed. His creatinine levels were stable at approximately 1.6 mg/dL (nadir: 1.49 mg/dL); however, there were no side effects, such as viral infections. The patient showed no complications or immunosuppression on follow-up after 2 years.

## 3. Discussion

Urological complications are the most common surgical complications of kidney transplantation. These mainly involve leakage from the ureter, resulting in the formation of urinomas. This complication causes abdominal pain, swelling of the transplant site, elevated serum creatinine level, oliguria, and/or signs of systemic infection [1]. Urine leakage usually occurs from the distal regions of the ureter in the early posttransplantation period (7–10 days), and its incidence rate ranges from approximately 1.5 to 6.0% [1]. Although native ureters receive blood supply from both renal arteries and collaterals, the graft ureter only depends on the arterial and venous supply from the renal vessels and lower polar branches. The periureteric tissue, also called the “golden triangle,” must be preserved by minimal dissection [1, 2]. Preserving the periureteral tissue and maintaining adequate length without tension are considered important factors for avoidance of ischemia and/or necrosis of the ureter that could cause rupture. In our case, we assumed that the ureteral leakage was caused by (i) excessive dissection of the periureteral tissues during donor nephrectomy resulting in insufficient blood supply and thus necrosis of the ureteral wall, (ii) absence of a ureteral stent, and (iii) edema of the orifice caused by elevated hydraulic pressure in the graft ureter, leading to the rupture.

In contrast, deficiency of FXIII could be congenital or acquired. In most surgical cases, FXIII deficiency is acquired owing to decreased synthesis and increased consumption of FXIII [4]. FXIII is a tetrameric molecule composed of 2 A-subunits of 83.2 kDa and 2 B-subunits of 79.7 kDa, which are held together noncovalently in a heterotetramer of 325.8 kDa. FXIIIa (thrombin-activated factor XIII) stabilizes fibrin through an amide or isopeptide bond that ligates adjacent fibrin monomers [5]. This is one of the main contributors of the clotting cascade; however, it also has an important role in wound healing and tissue repair. In ABO-incompatible kidney transplantation, double-filtration plasma pheresis (DFPP) is performed multiple times according to the anti-blood-type antibodies; however, DFPP can lead to reduction in fibrinogen and FXIII levels prior to surgery [6]. Because our patient underwent preemptive and ABO-compatible kidney transplantation, hemodialysis and DFPP were not conducted prior to the transplantation. Further, the donor was a dizygotic twin, and she was discharged without any complications. Further, decrease in the FXIII level 7 days after a major surgery [7], as in our case (the FXIII level was 48% at 8 days after the reconstruction procedure), has been suggested to be caused by consumption.

In our case, the drainage amount gradually decreased after the second operation, suggesting that neoanastomosis and ureteral stent placement were effective for the treatment of ureteral leakage. However, the efficacy of FXIII product administration remains unclear. FXIII administration is associated with adverse drug reactions, including hypersensitivity reactions, thromboembolic events, and viral infections. Solomon et al. showed 75 cases of adverse events owing to FXIII product administration using 20-year data [8]. Further, there is scarce evidence of its safety in patients with renal failure and those undergoing transplantation. Two reports showed the effectiveness and safety of FXIII administration in stem cell transplant recipients [9, 10]; however, the outcomes of such transfusion in kidney transplant recipients have not been established. Continuous leakage could contribute to surgical site infections and perinephric abscesses [11], causing graft loss. In addition, long-term complications could adversely affect the patient not only physically but also mentally. Although further studies are needed, administration of FXIII could be a therapeutic option for refractory urine leakage after transplantation. Because this is a description of a single case with a short follow-up duration, further accumulation of similar cases would be required to validate our findings.

Altogether, patients with a low FXIII level sometimes experience refractory postoperative fistulas; however, scarce related data exist with respect to kidney transplant patients. This is the first case reporting on the success of transfusion of FXIII products for the management of transplantation-associated complications without any adverse effects using an appropriate immunosuppression regimen. Further studies are warranted to confirm the efficacy of the treatment modality proposed herein.
